# Prevalence and risk factors for postoperative atrial fibrillation following pulmonary resection: a systematic review and meta-analysis

**DOI:** 10.1186/s13019-026-04093-x

**Published:** 2026-04-18

**Authors:** Yuping Yang, Guoqiang Zhong

**Affiliations:** Department of Cardiology, First Affiliated Hospital of Guangxi Medical University, Guangxi Zhuang Autonomous Region, Shuangyong Road 6, Nanning, 530021 People’s Republic of China

**Keywords:** PR, POAF, Prevalence, Risk factors, Meta-analysis

## Abstract

**Objective:**

This study aimed to perform a meta-analysis integrating multiple sources to investigate risk factors for postoperative atrial fibrillation (POAF) in patients undergoing pulmonary resection (PR).

**Methods:**

We searched English databases (PubMed, Cochrane Library, Embase, Web of Science (WOS)) and Chinese databases (China National Knowledge Infrastructure (CNKI), Wanfang Data, VIP Database, Sinomed) up to September 25, 2025. Observational studies utilizing multivariate logistic regression to analyze the prevalence and risk factors of atrial fibrillation (AF) following PR were selected. Meta-analysis was conducted in accordance with Preferred Reporting Items for Systematic Reviews and Meta-Analyses (PRISMA) guidelines. Data were extracted, and pooled odds ratios (OR) with 95% confidence intervals (CI) were calculated using STATA 15.0.

**Results:**

Eighteen studies involving 25,238 patients were included, among whom 1,967 developed POAF, yielding a prevalence of 7.79%. Meta-analysis revealed significant associations between POAF and the following risk factors: Age (OR = 1.04, 95% CI: 1.01–1.06, p = 0.011), Age ≥ 65 years (OR = 2.92, 95% CI: 2.02–4.23, p < 0.001), Male (OR = 1.93, 95% CI: 1.46–2.56, p < 0.001), Hypertension (OR = 1.34, 95% CI: 1.14–1.57, p < 0.001), Lymph node dissection (OR = 2.18, 95% CI: 1.12–4.24, p = 0.021), Thoracotomy (OR = 1.71, 95% CI: 1.40–2.09, p < 0.001), Lobectomy (OR = 2.68, 95% CI: 1.05–6.81, p = 0.039), Pneumonectomy (OR = 4.33, 95% CI: 2.12–8.84, p < 0.001), Operative time (minutes) (OR = 1.05, 95% CI: 1.01–1.09, p = 0.026), History of hyperthyroidism (OR = 5.67, 95% CI: 1.97–16.32, p < 0.001), Pre-existing atrial fibrillation (Pre-existing AF) (OR = 15.79, 95% CI: 4.69–53.13, p < 0.001), Red blood cell transfusion (OR = 2.78, 95% CI: 2.22–3.47, p < 0.001). No significant association was found with video-assisted thoracoscopic surgery (VATS) (OR = 1.02, 95% CI: 0.29–3.61, p = 0.97).

**Conclusion:**

This study suggests that POAF following PR is associated with multiple patient- and surgery-related risk factors, many of which are identifiable preoperatively. Pre-existing AF was identified as the strongest predictor among the factors analyzed. These findings support the rationale for integrating preoperative risk assessment into the planning of individualized postoperative monitoring and prevention strategies, which may help improve clinical outcomes.

**Supplementary Information:**

The online version contains supplementary material available at 10.1186/s13019-026-04093-x.

## Introduction

POAF is defined as new-onset AF occurring within 30 days after surgery. It represents the most common arrhythmia following PR, with a reported incidence of 10% to 40%. This risk is particularly heightened in elderly patients and those undergoing lobectomy or pneumonectomy. The incidence of POAF typically peaks 2 to 4 days after surgery. While most episodes are paroxysmal, approximately 30% may progress to persistent AF [1–4].

The pathophysiology of POAF differs from that of general AF. It involves distinct mechanisms triggered by the surgical context, including trauma-induced inflammation, sympathetic nervous system activation, oxidative stress, electrolyte imbalances, and cardiac autonomic dysfunction [5–7]. Additionally, physiological consequences of lung resection—such as reduced lung volume, increased pulmonary vascular resistance, and elevated right ventricular afterload—may further contribute to POAF development [8]. Clinically, POAF is significant because it not only prolongs hospital stay by 1–3 days and increases healthcare costs but is also associated with serious complications. These include hemodynamic instability, thromboembolic events (especially stroke), heart failure, and potentially reduced long-term survival [9–12].

PR is a primary surgical approach for treating lung cancer, benign pulmonary tumors, tuberculosis, and other lung diseases. Based on the extent of resection, it can be classified into wedge resection, segmentectomy, lobectomy, pneumonectomy, and bronchial sleeve lobectomy [13–15] (Figure S1). With advances in thoracic surgery, the procedure has evolved from traditional open thoracotomy to minimally invasive techniques such as VATS and robot-assisted surgery, significantly reducing surgical trauma and postoperative complications [16–19]. Nevertheless, PR is still associated with various complications, among which POAF remains one of the most frequent arrhythmias, considerably impacting patients’ postoperative recovery and long-term outcomes.

Numerous studies have reported risk factors for POAF after PR. Previous literature suggests that patient age [20, 21], gender [22, 23], history of hypertension [24, 25], surgical approach [20, 26, 27], intraoperative blood transfusion [24, 25], and neoadjuvant therapy [20, 25] may be associated with POAF occurrence. However, findings across studies are inconsistent, and individual studies are often limited by sample size and statistical power, making it difficult to comprehensively evaluate the true relationship between these risk factors and POAF. Moreover, the magnitude of association varies among studies, and the roles of certain factors—such as the extent of lymph node dissection [28, 29] and the use of VATS [20, 26] —remain controversial. Additionally, the spectrum of POAF risk factors may evolve with advancements in surgical techniques and perioperative management.

Several reviews have summarized risk factors for POAF following PR [30–33]. The present meta-analysis addresses a key methodological gap by synthesizing evidence exclusively from studies that employed multivariate logistic regression analysis. This approach provides adjusted effect estimates that are more robust against confounding, offering a clearer and more reliable hierarchy of independent risk factors compared to narrative reviews or meta-analyses of unadjusted data [34]. It specifically aims to resolve inconsistencies in the literature where the independent effect of certain factors (e.g., lymph node dissection, surgical approach) remains debated [28]. Therefore, this study aims to systematically search databases including PubMed, Cochrane Library, Embase, WOS, CNKI, Wanfang Data, VIP Database, and Sinomed to identify studies employing multivariate logistic regression analysis. A meta-analysis will be conducted to synthesize evidence on risk factors for POAF following PR, thereby providing more reliable evidence for clinical practice. The findings may aid in identifying high-risk patients, formulating individualized prevention strategies, and ultimately improving patient outcomes and healthcare quality.

## Methods

### Information sources and literature search

This systematic review and meta-analysis was conducted and reported in accordance with the PRISMA statement and the Meta-analysis of Observational Studies in Epidemiology (MOOSE) guidelines to ensure standardization and transparency [35, 36]. The study protocol was registered in the International Prospective Register of Systematic Reviews (IPRSR) (PROSPERO: CRD420251071300). Ethical approval and informed consent were not required, as this study did not involve direct research on human participants. Two investigators independently performed a systematic literature search using the following databases: PubMed, Cochrane Library, Embase, WOS, CNKI, Wanfang Data, VIP Database, and Sinomed. The search included studies published up to September 25, 2025. The Chinese search terms were “肺切除术” AND “房颤” AND “危险因素”. The English search terms were “lung surgery” AND “Atrial Fibrillation” AND “Risk Factors”. Additionally, we manually reviewed published systematic reviews and reference lists of included studies. It is important to note that our search strategy was primarily focused on published, peer-reviewed literature. We did not conduct a systematic search of grey literature sources (e.g., dissertations, trial registries, conference proceedings) beyond the aforementioned database searches and manual review of references. The search strategy was designed based on the Participants, Interventions, Comparisons, and Outcomes (PICO) framework (Table S1 and S2 in the Supplement). The PICO framework was applied as follows to structure the research question and guide the search: Participants(P): Adult patients (≥ 18 years) undergoing PR for any indication. Intervention/Exposure(I): Prespecified patient-related, disease-related, and surgery-related factors hypothesized or reported as risks for POAF (e.g., advanced age, male gender, specific surgical approaches like thoracotomy or VATS, extent of resection, lymph node dissection). Comparison(C): Patients not exposed to the specific risk factor under investigation (e.g., younger patients, females, patients undergoing sublobar resection without lymph node dissection). Outcome(O): The primary outcome was the occurrence of new-onset POAF within 30 days after PR, as defined by the individual studies. This framework underpinned the development of the key search terms and their synonyms to ensure a comprehensive and targeted literature retrieval.

### Inclusion and exclusion criteria

#### Inclusion criteria

A. Patients who developed POAF after PR; b. Studies using multivariate logistic regression to analyze risk factors for POAF; c. Officially published journal articles; d. Observational studies (cohort, case–control, cross-sectional); e. Sufficient data from two or more outcomes for statistical analysis.

#### Exclusion criteria

A. Duplicate publications, conference abstracts, reviews, meta-analyses, case reports, studies unrelated to the topic, or those lacking detailed data; b. Studies that did not employ multivariate logistic regression to assess POAF risk factors.

### Quality assessment and data extraction

Two reviewers (Yuping Yang, Guoqiang Zhong) independently assessed study quality. Cohort and case–control studies were evaluated using the Newcastle–Ottawa Scale (NOS), with scores ranging from 0 to 9. Studies scoring 0–4, 5–6, and 7–9 were classified as low, moderate, and high quality, respectively [37]. Studies with NOS scores ≥ 6 were included. Cross-sectional studies were assessed using the Agency for Healthcare Research and Quality (AHRQ) criteria, with scores of 0–3, 4–7, and 8–11 indicating low, moderate, and high quality, respectively [38]. To quantify inter-reviewer agreement, Cohen’s kappa (κ) statistic was calculated for both the study selection process and the quality assessment (NOS scoring) [39]. The results indicated substantial agreement in both phases (selection: κ = 0.063; quality: κ = 0.072). Any persisting discrepancies were then resolved by a third reviewer. Extracted data included: first author, sample size, country/region, publication year, number of POAF and non-POAF cases, POAF prevalence, study design, and risk factors for POAF (e.g., age, age ≥ 65, gender, hypertension, lymph node dissection, thoracotomy, VATS, lobectomy, pneumonectomy, operative time (minutes), history of hyperthyroidism, pre-existing AF, red cell transfusion, etc.). Data were independently extracted by two reviewers.

### Statistical analysis and outcome measures

Statistical analyses were performed using Stata 15.0. OR with 95% CI were used as effect measures for categorical data. Heterogeneity was assessed using the Q-test and I^2^ statistic. A fixed-effects model was applied if P ≥ 0.1 and I^2^ ≤ 50%; otherwise, a random-effects model was used. Sensitivity analysis was conducted by alternating effect models and sequentially excluding studies. Further, to assess the robustness of our findings and the impact of missing data, we performed additional sensitivity analysis. Specifically, for the meta-analysis of age as a risk factor, we excluded studies that did not report relevant data (indicated as “NR” in Table 1) to evaluate their influence on the pooled estimate. Publication bias was evaluated using Egger’s or Begg’s test under the random-effects model. A two-tailed p < 0.05 was considered statistically significant.

**Table 1:** Characteristics of included literature studies

Author	Year	Country	Age(years)		Gender (Male/Female)		Prevalence rate（%）	Study design	NOS/AHRQ
			POAF	N-POAF	POAF	N-POAF			
DD Hollings [20]	2010	USA	74.70±8.90	65±12.80	33/32	121/174	18.05%	Case-control study	7
Y Muranishi [28]	2017	Japan	70.40 (49–86)	67.30 (31–85)	22/16	328/227	6.40%	Case-control study	7
CY Tong [24]	2024	China	NR	NR	230	8487	6.00%	Cohort study	7
D Ai [41]	2014	China	67.72 ± 9.030	63.96 ± 10.74	88/48	289/278	19.34%	Case-control study	7
SH Lee [25]	2016	Korea	65 ±9.20	59.30 ±11.70	459/96	2425/1682	11.90%	Cohort study	7
T Hayashi [26]	2024	Japan	NR	NR	50/12	552/341	6.49%	Case-control study	8
Y Han [22]	2024	China	70.90±4.40	70.10±4.30	92/61	1253/1514	5.23%	Cohort study	7
LF Zhang [42]	2023	China	64.04 ± 7.42	59.99 ± 9.03	66/6	96/48	33.33%	Case-control study	7
V Crispi [27]	2022	UK	70±8.20	69 (62–74)	49/52	595/671	7.38%	Cross-sectional study	9
M Kashiwagi [29]	2023	Japan	73 (69–77)	70 (64–76)	33/13	769/496	3.50%	Case-control study	8
V Scheggi [21]	2023	Italy	75 (74–79)	68 (67–70)	24/11	173/130	10.35%	Case-control study	8
Qian JK [43]	2025	China	NR	NR	24/14	36/26	38.00%	Case-control study	8
Gong D [44]	2022	China	61 (59, 72)	58 (53, 65)	11/4	284/476	1.81%	Cohort study	8
Huang XF [45]	2022	China	64.26±8.60	55.95±11.17	78/67	368/459	14.91%	Case-control study	8
Zhou F [46]	2018	China	NR	NR	88/44	97/99	40.24%	Case-control study	8
Zheng M [47]	2016	China	73±7	70±7	34/16	92/84	22.12%	Case-control study	8
Wang YD [48]	2014	China	NR	NR	52	310	14.36%	Case-control study	8
Jiang YJ [49]	2023	China	68.50(63,74)	68(60,73)	27/15	126/45	19.71%	Case-control study	8

POAF = Post-operative atrial fibrillation, NR = No reported, N-POAF = No post-operative atrial fibrillation, NOS = Newcastle–ottawa scale. AHRQ = agency of healthcare research and quality

## Results

### Literature search overview

**Fig. 1:**
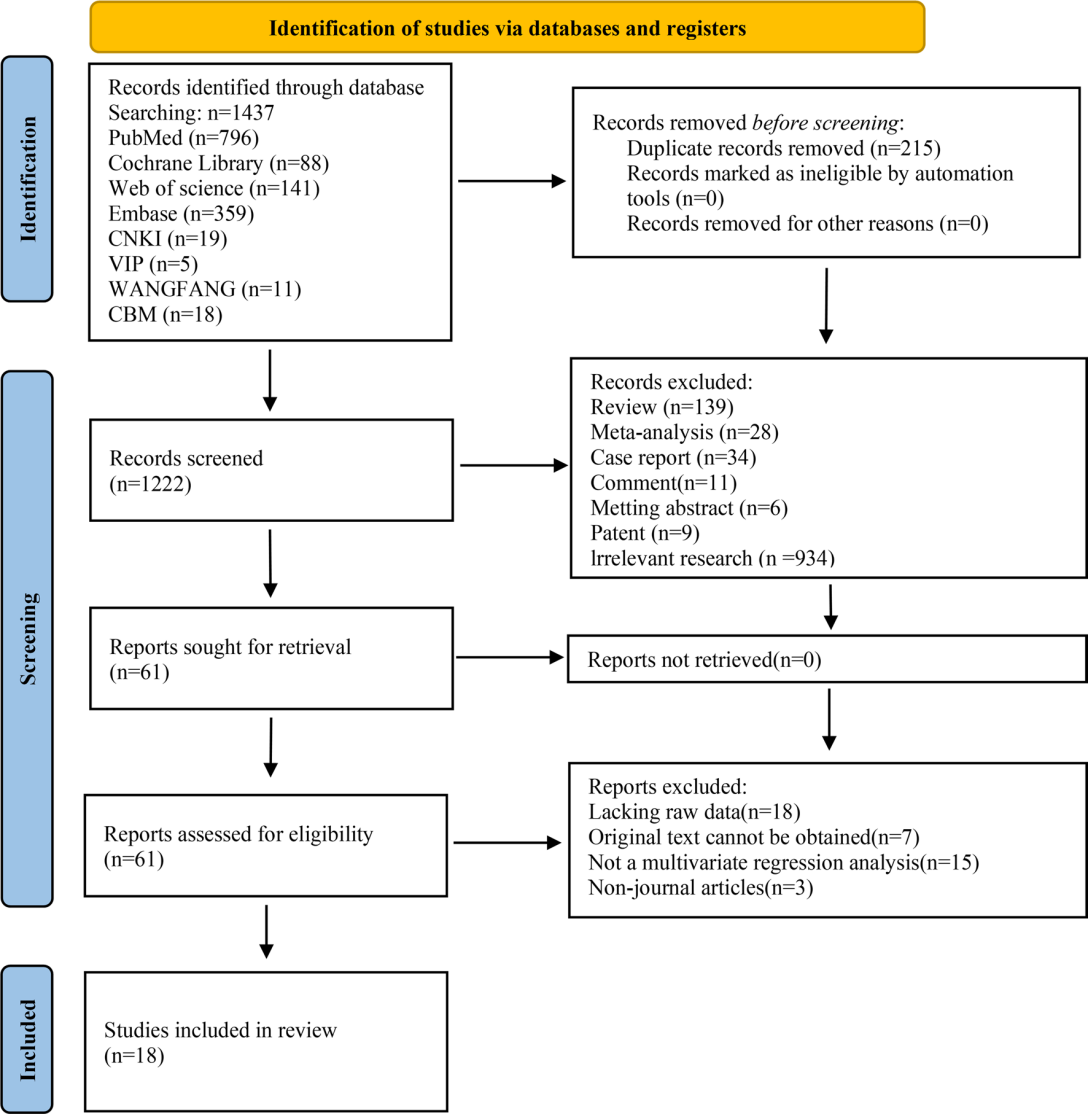
Process and outcomes of literature screening. This diagram illustrates the sequential process of identifying, screening, and selecting eligible studies according to the PRISMA guidelines. A total of 1437 records were retrieved from major electronic databases including PubMed (n = 796), Cochrane Library (n = 88), WOS (n = 141), Embase (n = 359), CNKI (n = 19), WIP (n = 5), Wanfang Data (n = 11), and CBM (n = 18). After removing duplicates, 1222 records underwent title and abstract screening, during which 139 reviews, 28 meta-analyses, 34 case reports, 11 comments, 6 meeting abstracts, 9 patents, and 934 irrelevant studies were excluded, leaving 61 potentially eligible articles. Following full-text assessment, 18 studies met inclusion criteria and were included in the quantitative synthesis (meta-analysis)

Based on the search strategy, 1,437 publications were initially identified. After removing duplicates, the titles and abstracts of the remaining 1,222 studies were screened. Subsequently, 61 studies were deemed eligible for full-text review. Among these, 43 studies were excluded due to not meeting the inclusion criteria. Finally, 18 studies were included in this systematic review and meta-analysis, comprising 11 science citation indexed (SCI) articles [20–22, 24–27, 29, 40–42] and 7 articles from Chinese journals [43–49]. The study selection process is summarized in Fig. 1.

### Characteristics of included studies

A total of 18 studies involving 25,238 patients were included, among whom 1,967 developed POAF, yielding an incidence rate of 7.79%. Eleven studies were conducted in China [22, 24, 41–49], three in Japan [26, 29, 40], and one each in South Korea [25], the USA [20], the UK [27], and Italy [21]. The included studies were published between 2010 and 2025. The mean age of the patients was approximately 69.04 years. The mean NOS score of the included studies was 7.59. Seven studies [20, 22, 24, 25, 40–42] scored 7, ten studies [21, 26, 29, 43–49] scored 8, and the included cross-sectional study [27] received an AHRQ score of 9, indicating that all were high-quality studies. (Table 1). The consistency among the reviewers was tested by Cohen’s kappa and showed a high degree of consistency in both the literature screening (κ = 0.63) and quality assessment (κ = 0.72) stages, indicating that the data extraction process was reliable.

### Age and age ≥ 65

**Fig. 2:**
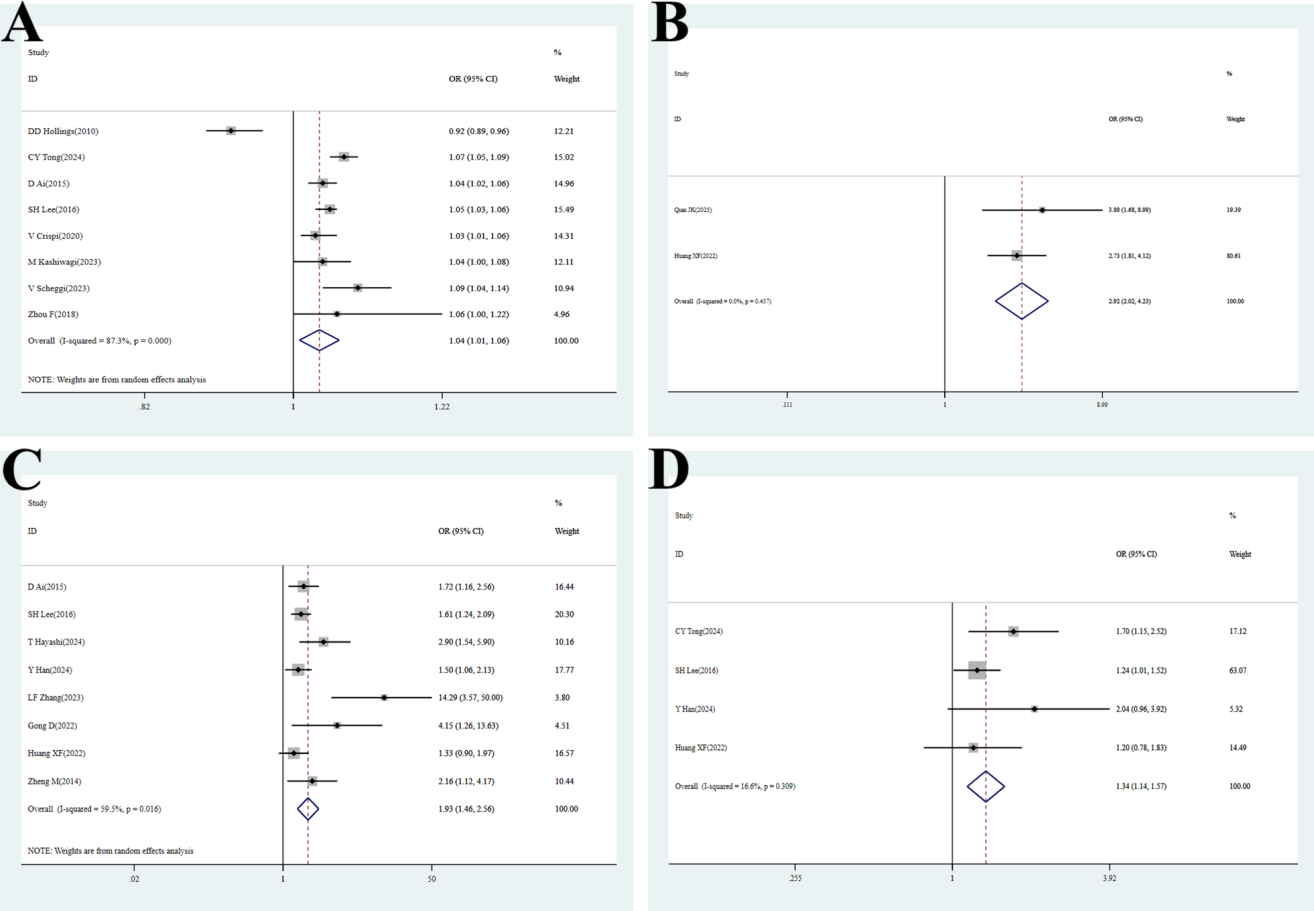
Forest plots of meta-analyses for risk factors and outcomes. ​​(**A**) Age:​​ Pooled OR: 1.04 (95% CI: 1.01–1.06), indicating a statistically significant association between increased age and POAF risk. High heterogeneity was observed (I^2^ = 87.3%, p < 0.001). ​​(**B**) Age ≥ 65 years:​​ Pooled OR: 2.92 (95% CI: 2.02–4.23), demonstrating a significantly elevated risk in older patients. Heterogeneity was very high (I^2^ = 0, p = 0.457). ​​(**C**) Gender:​​ Pooled OR: 1.93 (95% CI: 1.46–2.56), suggesting male as a risk factor. Moderate heterogeneity was present (I^2^ = 59.5%). ​​(**D**) Hypertension:​​ Pooled OR: 1.34 (95% CI: 1.14–1.57), confirming hypertension as a significant risk factor with low heterogeneity (I^2^ = 16.6%, p = 0.309)

Eight studies [20, 21, 24, 25, 27, 29, 41, 46] examined the association between age and POAF after PR. Significant heterogeneity was observed among studies (P < 0.001, I^2^ = 87.3%). Random-effects model analysis [OR = 1.04, 95% CI (1.01–1.06), P = 0.011] indicated that each one-year increase in age was associated with a 4% higher risk of POAF, confirming age as an independent risk factor (Fig. 2A, Table 2). Given the high heterogeneity(I^2^ ≥ 50%), sensitivity analysis confirmed the robustness of the result. Publication bias tests (Begg’s test P = 0.805; Egger’s test P = 0.511) suggested a low likelihood of publication bias (Figure S2A, Figure S3A, Table 2, Table S3). However, conducted after excluding the two studies [24], [46] that did not report age data, yielded a consistent and robust result. This confirms that the overall conclusion is not substantially influenced by the absence of data from these studies (Figure S4).

**Table 2:** Detailed data on 12 inclusion factors for the POAF after lung surgery

Inclusion factors	Literatures(n)	Heterogeneity test		Publication bias		Effect model	OR (95% CI)	P-value	Reference
		P-value	I^2^%	Begg	Egger				
**Age**	8	< 0.001	87.3	0.805	0.511	Random effects	1.04(1.01–1.06)	0.011*	[20, 21, 24, 25, 27, 29, 41, 46]
**Age ≥ 65**	2	0.457	0	-	-	Fixed effects	2.92(2.02–4.23)	< 0.001*	[43, 45]
**Gender**	8	0.016	59.5	0.013	0.006	Random effects	1.93(1.46–2.56)	< 0.001*	[22, 25, 26, 41, 42, 44, 45, 47]
**Hypertension**	4	0.309	16.6	-	-	Fixed effects	1.34(1.14–1.57)	< 0.001*	[22, 24, 25, 45]
**Lymph node dissection**	7	0.003	69.3	0.652	0.356	Random effects	2.18(1.12–4.24)	0.021*	[26, 29, 40, 42, 45, 48, 49]
**Thoracotomy**	5	0.332	12.8	-	-	Fixed effects	1.71(1.40–2.09)	< 0.001*	[21, 25, 27, 40, 49]
VATS	2	0.024	80.3	0.317	#	Random effects	1.02(0.29–3.61)	0.97	[20, 26]
**Lobectomy**	3	0.034	70.5	0.602	0.649	Random effects	2.68(1.05–6.81)	0.039*	[26, 27, 45]
**Pneumonectomy**	3	0.988	0	-	-	Fixed effects	4.33(2.12–8.84)	< 0.001*	[27, 43, 49]
**Operative time (minutes)**	3	< 0.001	92.0	0.117	0.254	Random effects	1.05(1.01–1.09)	0.026*	[24, 25, 43]
**History of hyperthyroidism**	2	0.596	0	-	-	Fixed effects	5.67(1.97–16.32)	< 0.001*	[22, 29]
**Pre-existing AF**	2	0.853	0	-	-	Fixed effects	15.79(4.69–53.13)	< 0.001*	[20, 49]
**Red cell transfusion**	2	0.508	0	-	-	Fixed effects	2.78(2.22–3.47)	< 0.001*	[24, 25]

POAF: Post-operative atrial fibrillation, VATS: Video-assisted thoracoscopic surgery; Begg: P value for Begg test; Egger: P value for Egger test; P value for begg test or egger test ≥ 0.05, indicates that there is no significant publication bias; Conversely, it indicates that there is statistically publication bias; “-”: Indicates that there is no need to calculate publication bias due to low heterogeneity; #: It indicates that the Egger test cannot be conducted due to the inclusion of less than three literatures; * indicates P < 0.05, indicating that this factor is a risk factor for POAF

Additionally, two studies [43, 45] specifically evaluated the association between age ≥ 65 and POAF. Subgroup analysis showed no significant heterogeneity (P = 0.457, I^2^ = 0). Fixed-effects model analysis [OR = 2.92, 95% CI (2.02–4.23), P < 0.001] indicated that patients aged ≥ 65 had a 2.92-fold higher risk of POAF compared to younger patients (Fig. 2B, Table 2).

### Gender

Eight studies [22, 25, 26, 41, 42, 44, 45, 47] involving 6,237 male and 5,132 female patients analyzed the effect of gender on POAF. Significant heterogeneity was detected (P = 0.016, I^2^ = 59.5%). Random-effects model analysis [OR = 1.93, 95% CI (1.46–2.56), P < 0.001] indicated that males had a 1.93-fold higher risk of POAF than females, confirming male as an independent risk factor **(**Fig. 2C**, **Table 2**)**. Sensitivity analysis supported the robustness of the result. Significant publication bias was suggested (Begg’s test P = 0.013; Egger’s test P = 0.006) (Figure S2B, Figure S3B, Table 2, Table S3).

### Hypertension

Four studies [22, 24, 25, 45] evaluated the impact of hypertension on POAF. Low heterogeneity was observed (P = 0.309, I^2^ = 16.6%). Fixed-effects model analysis [OR = 1.34, 95% CI (1.14–1.57), P < 0.001] indicated a 34% increased risk of POAF in hypertensive patients, confirming hypertension as a significant risk factor, potentially related to atrial structural and electrophysiological remodeling **(**Fig. 2D**, **Table 2**)**.

### Lymph node dissection

**Fig. 3:**
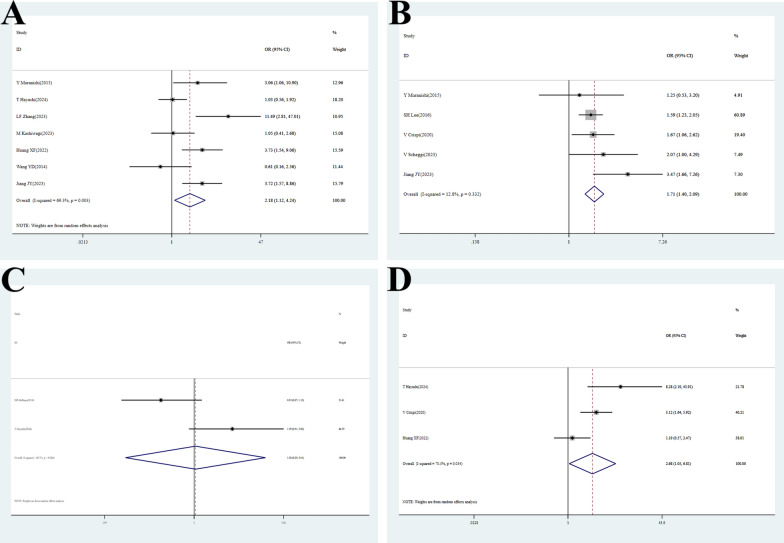
Forest plots of meta-analyses for risk factors and outcomes. ​​(**A**) Lymph node dissection:​​ The overall pooled OR is 2.18 (95% CI: 1.12–4.24), indicating a statistically significant association. Significant heterogeneity was observed among the studies (I^2^ = 69.3%, p = 0.003). ​​(**B**) Thoracotomy: The overall pooled OR is 1.71 (95% CI: 1.40–2.09), indicating a statistically significant association. Low heterogeneity was observed among the studies (I^2^ = 12.8%). (**C**) VATS: The overall pooled OR is 1.02 (95% CI: 0.29–3.61, P = 0.970), indicating a no statistically significant association. (**D**) Lobectomy: The overall pooled OR is 2.68 (95% CI: 1.05–6.81), indicating a statistically significant association

Seven studies [26, 29, 40, 42, 45, 48, 49] reported on the impact of lymph node dissection on POAF. Significant heterogeneity was present (P = 0.003, I^2^ = 69.3%). Random-effects model analysis [OR = 2.18, 95% CI (1.12–4.24), P = 0.021] indicated that patients undergoing lymph node dissection had a 2.18-fold higher risk of POAF **(**Fig. 3A**, **Table 2**)**. Sensitivity analysis confirmed result stability. Publication bias was unlikely (Begg’s test P = 0.652; Egger’s test P = 0.356) (Figure S2C, Figure S3C, Table 2, Table S3).

### Thoracotomy

Five studies [21, 25, 27, 40, 49] analyzed the effect of thoracotomy on POAF. No significant heterogeneity was detected (P = 0.332, I^2^ = 12.8%). Fixed-effects model analysis [OR = 1.71, 95% CI (1.40–2.09), P < 0.001] indicated a 71% increased risk of POAF in patients undergoing thoracotomy, confirming it as a significant risk factor, potentially due to greater surgical trauma and postoperative pain (Fig. 3B, Table 2).

### VATS

Two studies [20, 26] evaluated the effect of VATS on POAF. Significant heterogeneity was observed (P = 0.024, I^2^ = 80.3%). Random-effects model analysis [OR = 1.02, 95% CI (0.29–3.61), P = 0.970] suggested no significant association between VATS and POAF (Fig. 3C, Table 2). However, this conclusion is based on only two studies and is accompanied by considerable heterogeneity (I^2^ = 80.3%). Therefore, it should be interpreted with extreme caution and considered preliminary. Sensitivity analysis supported robustness. Publication bias was unlikely (Begg’s test P = 0.317) (Figure S2D, Figure S3D, Table 2, Table S3).

### Lobectomy

Three studies [26, 27, 45] analyzed the impact of lobectomy on POAF. High heterogeneity was present (P = 0.034, I^2^ = 70.5%). Random-effects model analysis [OR = 2.68, 95% CI (1.05–6.81), P = 0.039] indicated that patients undergoing lobectomy had a 2.68-fold higher risk of POAF compared to those receiving sublobar resection, confirming lobectomy as a strong risk factor, possibly due to larger surgical extent and greater cardiac mechanical stimulation (Fig. 3D, Table 2). Sensitivity analysis confirmed robustness. Publication bias was unlikely (Begg’s test P = 0.602; Egger’s test P = 0.649) (Figure S2E, Figure S3E, Table 2, Table S3).

### Pneumonectomy

**Fig. 4:**
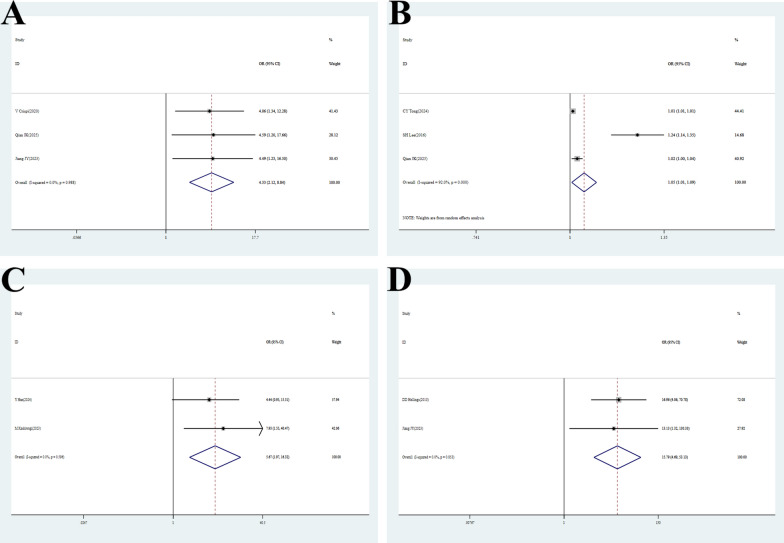
Forest plots of meta-analyses for risk factors and outcomes.​​ (**A**) Pneumonectomy: Forest plot of studies evaluating pneumonectomy as a risk factor. The overall pooled OR was 4.33 (95% CI: 2.12–8.84), indicating a statistically significant association. No significant heterogeneity was observed (I^2^ = 0, p = 0.988). (**B**) Operative time (minutes): Forest plot of studies evaluating operative time as a risk factor. The overall pooled OR was 1.05 (95% CI: 1.01–1.09), indicating a statistically significant association. Significant heterogeneity was observed (I^2^ = 92.0%, p < 0.001). (**C**) History of hyperthyroidism: Forest plot of studies evaluating history of hyperthyroidism as a risk factor. The overall pooled OR was 5.67 (95% CI: 1.97–16.32), indicating a statistically significant association. No significant heterogeneity was observed (I^2^ = 0, p = 0.596). (**D**) Pre-existing AF: Forest plot of studies evaluating pre-existing atrial fibrillation as a risk factor. The overall pooled OR was 15.79 (95% CI: 4.69–53.13), indicating a statistically significant association. No significant heterogeneity was observed (I^2^ = 0, p = 0.853)

Three studies [27, 43, 49] evaluated the effect of pneumonectomy on POAF. Low heterogeneity was observed (P = 0.988, I^2^ = 0). Fixed-effects model analysis [OR = 4.33, 95% CI (2.12–8.84), P < 0.001] indicated a 33% increased risk of POAF in patients undergoing pneumonectomy, confirming it as a risk factor (Fig. 4A, Table 2).

### Operative time (minutes)

Three studies [24, 25, 43] analyzed the impact of operative time on POAF. High heterogeneity was present (P < 0.001, I^2^ = 92.0%). Random-effects model analysis [OR = 1.05, 95% CI (1.01–1.09), P = 0.026] indicated that each additional minute of operative time increased the risk of POAF by 5% **(**Fig. 4B**, **Table 2**)**. Sensitivity analysis confirmed robustness. Publication bias was unlikely (Begg’s test P = 0.117; Egger’s test P = 0.254) (Figure S2F, Figure S3F, Table 2, Table S3).

### History of hyperthyroidism

Two studies [22, 29] evaluated the impact of a history of hyperthyroidism on POAF. Low heterogeneity was observed (P = 0.596, I^2^ = 0). Fixed-effects model analysis [OR = 5.67, 95% CI (1.97–16.32), P < 0.001] indicated that patients with a history of hyperthyroidism had a 5.67-fold higher risk of POAF **(**Fig. 4C**, **Table 2**)**.

### Pre-existing AF

Two studies [20, 49] analyzed the effect of pre-existing AF on POAF. Low heterogeneity was present (P = 0.853, I^2^ = 0). Fixed-effects model analysis [OR = 15.79, 95% CI (4.69–53.13), P < 0.001] indicated that patients with pre-existing AF had a 15.79-fold higher risk of postoperative AF recurrence **(**Fig. 4D**, **Table 2**)**.

### Red cell transfusion

**Fig. 5:**
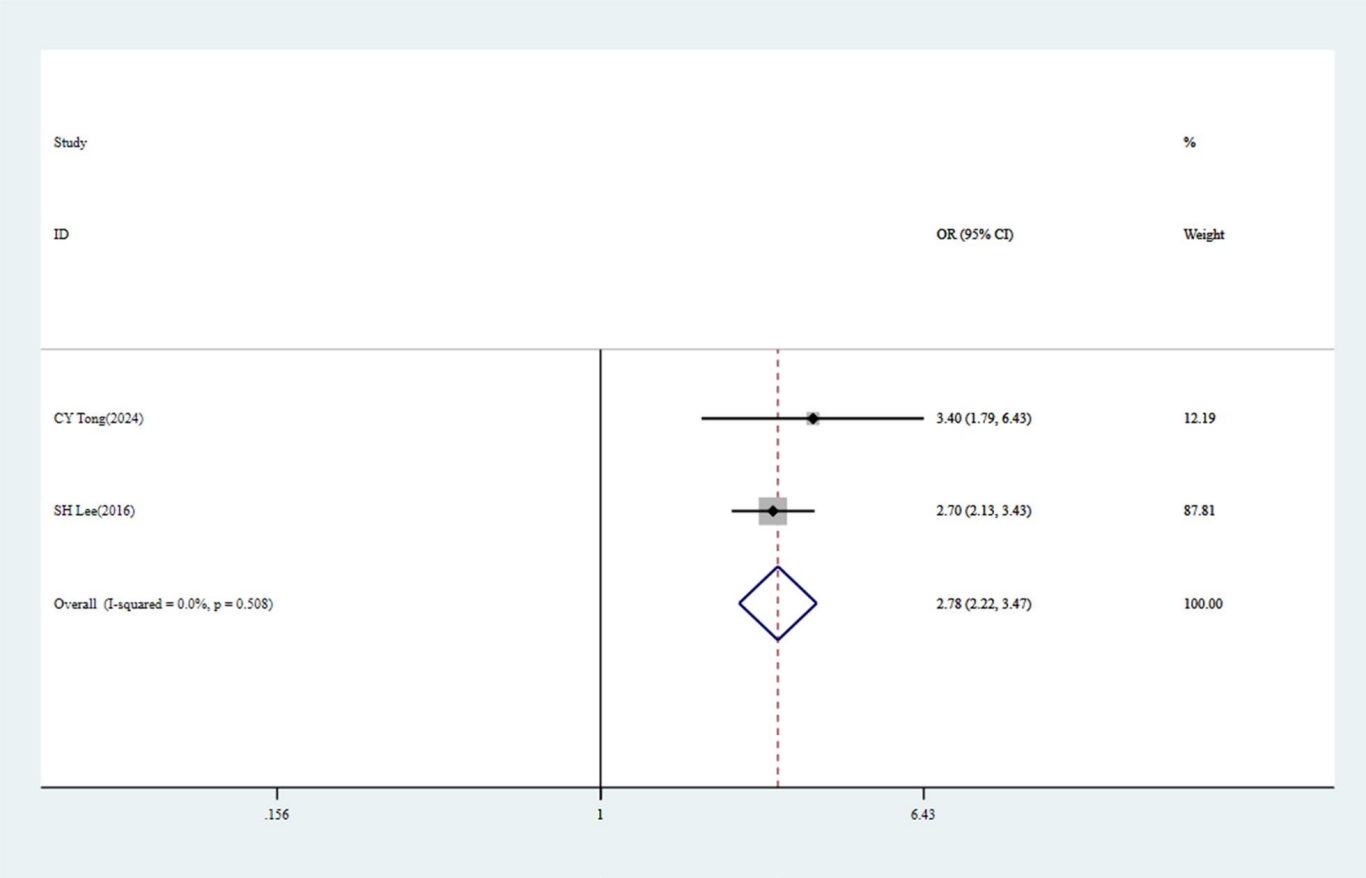
Forest plots of meta-analyses for risk factors and outcomes. Red Cell Transfusion: The overall pooled OR was 2.78 (95% CI: 2.22–3.47), indicating a statistically significant association. No heterogeneity was observed among the studies (I^2^ = 0, p = 0.508)

Two studies [24, 25] evaluated the impact of red cell transfusion on POAF. No heterogeneity was detected (P = 0.508, I^2^ = 0). Fixed-effects model analysis [OR = 2.78, 95% CI (2.22–3.47), P < 0.001] indicated that patients receiving red cell transfusion had a 2.78-fold higher risk of POAF, confirming it as a strong risk factor, potentially related to inflammatory response and increased volume load **(**Fig. 5**, **Table 2**)**.

In summary, this meta-analysis identified several significant risk factors for POAF. Based on the strength of evidence, these can be categorized as follows:

a. Very strong (OR ≥ 5): Pre-existing AF (OR = 15.79), History of hyperthyroidism (OR = 5.67). b. Strong (3 ≤ OR < 5): Pneumonectomy (OR = 4.33). c. Moderate (2 ≤ OR < 3): Age ≥ 65 years (OR = 2.92), Red cell transfusion (OR = 2.78), Lobectomy (OR = 2.68), Lymph node dissection (OR = 2.18). d. Low (1 ≤ OR < 2): Male (OR = 1.93), Thoracotomy (OR = 1.71), Hypertension (OR = 1.34), Operative time (OR = 1.05). e. Non-risk factor: VATS showed no significant association with POAF.

This descriptive categorization was based on the magnitude of the pooled OR, employing the following quantitative thresholds adapted from common interpretations in epidemiological research: Very strong association (OR ≥ 5), Strong association (3 ≤ OR < 5), Moderate association (2 ≤ OR < 3), and Low association (1 ≤ OR < 2) [50]. It is important to note that these thresholds serve as a simplified heuristic for summarizing the relative strength of evidence; the clinical interpretation of any risk factor must concurrently consider its confidence interval, biological plausibility, and the specific patient context.

## Discussion

This systematic review and meta-analysis integrated data from 18 observational studies, encompassing 25,238 patients undergoing PR, to comprehensively evaluate risk factors for POAF. The pooled analysis found an overall POAF incidence of 7.79%, which is consistent with previous reports [30–32]. We identified several significant risk factors and quantified their ORs, thereby clarifying their relative importance for perioperative risk stratification.

The development of POAF is understood to result from a complex interplay between patient-specific predispositions and surgery-related triggers. In the following sections, we first elaborate on the pathophysiological mechanisms underpinning the key risk factors identified. We then compare our findings with the existing body of evidence and conclude by discussing the clinical implications of our results.

### Pathophysiological mechanisms of major risk factors

#### Patient-related and non-modifiable factors

Advanced age (especially ≥ 65 years, OR = 2.92) and male (OR = 1.93) were moderate-low risk factors. Cardiac aging involves “atrial myopathy”, a progressive structural and functional deterioration of the atrial myocardium—characterized by interstitial fibrosis, disrupted gap junctions (e.g., Connexin 40), and mitochondrial dysfunction, leading to conduction heterogeneity and shortened effective refractory periods—ideal substrates for re-entrant arrhythmias [51, 52]. The sharp risk increase at age ≥ 65 may reflect cumulative damage and reduced physiological reserve. The higher risk in males may involve sex hormone effects (e.g., estrogen’s ion channel modulation, androgen-related fibrosis), sex-specific immune responses, and differences in atrial anatomy [53, 54].The strongest predictor was pre-existing AF (OR = 15.79), indicating profound electrophysiological and structural atrial remodeling. Perioperative stressors such as inflammation (elevated IL-6, TNF-α), sympathetic activation, and oxidative stress can easily trigger AF in this vulnerable substrate [55, 56]. Similarly, a history of hyperthyroidism (OR = 5.67) exerts direct genomic and non-genomic effects on atrial cardiomyocytes, including upregulation of L-type calcium channels and downregulation of potassium channels, shortening action potential duration and increasing automaticity. Electrophysiological remodeling may persist even after thyroid function normalizes [57, 58]. Hypertension (OR = 1.34) promotes left atrial enlargement and fibrosis via chronic pressure overload and renin-a​ngiotensin-aldosterone system (RAAS) activation, creating a pro-arrhythmic substrate [59, 60].

Special consideration of pre-existing AF as a risk factor, this meta-analysis identified pre-existing AF as the strongest risk factor for POAF following lung resection, with a pooled OR of 15.79 (95% CI: 4.69–53.13). While this finding is highly significant, its clinical interpretation requires careful consideration alongside several important limitations inherent in the available data.

First, regarding preoperative management, the two studies included in this analysis (Hollings et al. [20]; Jiang et al. [49]) did not report whether patients with pre-existing AF were on chronic anticoagulation or rhythm/rate control medications, or if such therapies were continued or paused perioperatively. This absence of data is a key limitation, as effective preoperative management may alter the underlying atrial substrate and potentially influence the risk of postoperative arrhythmia recurrence or exacerbation [61, 62].

Second, concerning the definition of “pre-existing AF”, both studies treated it as a binary variable (presence vs. absence) in their multivariate models. They did not provide stratified data based on AF type (e.g., paroxysmal vs. persistent), AF burden, or time since the last episode. The lack of this granularity prevents a nuanced analysis of how different AF phenotypes may confer varying levels of risk [63].

Finally, and most critically, is the challenge of distinguishing recurrence from de novo onset. The operational definition of POAF across all included studies was “new-onset AF within 30 days after surgery”. In clinical practice and within these studies, an atrial tachyarrhythmia occurring postoperatively in a patient with a known history of AF is typically counted as a POAF event. Pathophysiologically, a substantial proportion of these events likely represent a recurrence or exacerbation of the patient’s underlying arrhythmic condition, triggered by the potent stressors of surgery (e.g., inflammation, sympathetic activation, volume shifts), rather than a de novo arrhythmia in a previously unaffected atrium. This methodological overlap likely contributes significantly to the magnitude of the observed effect size (OR = 15.79), as it conflates recurrence risk with the risk of new incidence [64, 65].

Therefore, while this finding robustly identifies patients with pre-existing AF as the highest-risk group requiring intensive monitoring and strong consideration of prophylaxis, the estimated OR should be interpreted as a composite measure of “postoperative AF occurrence”. Future studies should aim to separately analyze true de novo POAF and AF recurrence, and report detailed preoperative management strategies to better refine risk prediction and enable tailored preventive measures for this distinct patient subgroup [66].

#### Surgery-related factors and traumatic stress

Surgical approach and extent were key determinants of POAF. Thoracotomy (OR = 1.71) causes greater tissue injury and pain than minimally invasive techniques, leading to stronger inflammatory and sympathetic responses. Peak levels of C-reactive protein (CRP) and IL-6 correlate with POAF incidence, and catecholamines shorten atrial refractory periods and promote ectopic activity [67, 68]. Notably, VATS was not significantly associated with POAF risk (OR = 1.02, P = 0.97). This suggests that the inherent physiological impact of lung resection itself may be a dominant driver of POAF risk, potentially outweighing the effect of the surgical approach (VATS vs. thoracotomy) in these studies. While VATS is established to reduce chest wall trauma and improve postoperative recovery, its specific role in mitigating POAF requires further investigation [69–71]. Given the limited number of studies (n = 2) and substantial heterogeneity, this finding is hypothesis-generating. It highlights a critical need for more high-quality, multivariate-adjusted studies to definitively assess the influence of surgical approach on POAF.

Larger resections—lobectomy (OR = 2.68) and pneumonectomy (OR = 4.33) —elevate risk through greater tissue injury, inflammation, mediastinal shift, cardiac rotation, and direct manipulation near pulmonary vein ostia, which are common AF triggers [72, 73]. Systematic lymph node dissection (OR = 2.18) is an increasingly recognized risk factor, likely due to injury or stimulation of pericardial and periesophageal autonomic ganglia. Autonomic imbalance (vagal suppression/sympathetic excess) is a key arrhythmogenic mechanism [74, 75]. However, it is important to acknowledge that this proposed autonomic mechanism, while plausible, remains speculative. The observed association could also be confounded by factors intrinsically linked to more extensive lymph node dissection, such as higher cancer stage (which may necessitate more radical dissection), longer operative time, and greater overall surgical complexity. These factors themselves are independent triggers for systemic inflammation, physiological stress, and subsequent arrhythmogenesis [76].

Prolonged operative time (OR = 1.05 per minute) serves as a proxy for surgical complexity and complications. Longer exposure to anesthesia, one-lung ventilation, and surgical stress amplifies inflammatory and oxidative damage [77–79]. Red cell transfusion (OR = 2.78) is a strong risk factor, not only due to volume overload but also transfusion-related immunomodulation (TRIM). Bioactive substances in stored blood activate neutrophils and monocytes, exacerbate inflammation, and impair endothelial function, promoting AF [80, 81].

### Comparison and integration with existing evidence

Our findings align with large international cohort studies and systematic reviews but provide more precise effect estimates through stricter inclusion criteria (multivariate regression studies) and an updated literature search. For example, our results on age and gender are consistent with analyses from databases like the s​ociety of thoracic surgeons (STS) [82–84], but we further quantified risk gradients for both continuous and categorical (≥ 65 years) age. (Table S4).

Regarding surgical approach, our study adds nuance to ongoing debates. While earlier studies suggested VATS might reduce POAF [85], recent high-quality propensity-matched studies support our conclusion that after adjusting for baseline characteristics, VATS and thoracotomy show similar POAF rates [27, 86, 87]. This highlights the importance of controlling for selection bias when comparing surgical techniques. (Table S4).

A key contribution of our study is the robust confirmation of factors previously underpowered or inconsistent in the literature. For instance, although lymph node dissection was mechanistically plausible, epidemiological evidence conflicted [33, 88]. By pooling 7 studies, we clearly identified it as an independent risk factor, aiding clinical trade-off between oncological radicality and arrhythmia risk. Critically, the identification of lymph node dissection as a risk factor should in no way discourage its performance when it is oncologically indicated. The primary goal remains achieving complete cancer resection and staging. The association highlighted here underscores the need for heightened awareness and potential prophylactic measures in patients undergoing this necessary procedure, rather than suggesting its avoidance [89]. Similarly, quantifying the risk of hyperthyroidism history—a rare but potent factor—has important clinical alert value. (Table S4).

### Clinical implications and practical recommendations

Based on our results, we propose a risk-stratified management framework for patients undergoing PR:

#### Preoperative risk assessment

Use standardized tools incorporating identified risk factors to stratify patients into low-, medium-, and high-risk groups. Focus prevention efforts on those with ≥ 65 years, male, hypertension, planned lobectomy/pneumonectomy with lymph node dissection. Those with pre-existing AF or hyperthyroidism are very high-risk.

#### Perioperative optimization

Intraoperative: Favor VATS where oncologically appropriate. Refine surgical techniques to minimize autonomic injury, reduce bleeding/transfusion, and improve efficiency [90, 91].

Postoperative: Extend Electrocardiogram (ECG) monitoring to at least post-op day 4 in medium/high-risk patients. For high-risk patients without contraindications, consider prophylactic beta-blockers (e.g., bisoprolol, metoprolol), supported by Randomized controlled trials (RCTs) [92, 93]. Amiodarone is an alternative but requires monitoring for side effects [94]. Aggressive pain control, electrolyte management (K⁺, Mg^2^⁺), and avoidance of fluid overload are essential [95].

The recommendation for beta-blocker prophylaxis should be guided by the latest 2024 ACC/AHA perioperative guideline, which provides a framework for individualized management based on patient-specific risk [96]. This guideline, along with supporting RCTs and meta-analyses, acknowledges the benefit of prophylaxis in reducing POAF incidence but emphasizes a nuanced application. Consistent with this guideline, the decision to initiate pharmacologic prophylaxis must be individualized, carefully weighing the demonstrated benefit (e.g., relative risk reduction) against potential risks. Key contraindications emphasized in the guideline, such as bradycardia, hemodynamic instability, active bronchospasm, or decompensated heart failure, must be strictly respected [96]. Therefore, prophylaxis with beta-blockers (or amiodarone as an alternative) should be considered only for patients identified as high-risk by this analysis and after a thorough assessment confirms the absence of contraindications and a favorable overall benefit-risk profile for the individual patient. **Cost-Effectiveness **

#### Considerations of Risk Stratification and Prophylaxis

Implementing a risk-stratified management approach based on the factors identified in this study holds promise not only for improving patient outcomes but also for optimizing healthcare resource utilization, suggesting favorable cost-effectiveness [97]. Accurate risk assessment allows for the targeted allocation of more resource-intensive interventions—such as extended ECG monitoring and prophylactic medications (e.g., beta-blockers, amiodarone) with their associated costs and potential side effects—to the subset of patients at genuinely high risk (e.g., those with multiple moderate-to-high risk factors, or very high-risk conditions like pre-existing AF or hyperthyroidism). Conversely, lower-risk patients can be spared unnecessary interventions and their attendant costs and risks. This “targeted prevention” model, compared to a “one-size-fits-all” strategy, has the potential to reduce the overall incidence of POAF while minimizing unnecessary healthcare expenditure and drug-related adverse events [98]. Future research should aim to formally quantify the cost-effectiveness of different prevention strategies derived from this risk profile by developing decision-analytic models that incorporate detailed, localized data on medical costs (e.g., costs associated with prolonged hospitalization due to POAF, stroke treatment, anticoagulation therapy) and patient outcomes.

### Consideration of heterogeneity

Substantial to considerable heterogeneity (I^2^ ≥ 50%) was observed for several key risk factors in this meta-analysis, including age (I^2^ = 87.3%), gender (I^2^ = 59.5%), lymph node dissection (I^2^ = 69.3%), video-assisted thoracoscopic surgery (VATS, I^2^ = 80.3%), lobectomy (I^2^ = 70.5%), and operative time (I^2^ = 92.0%). While we addressed this heterogeneity using a random-effects model and confirmed the robustness of the primary findings via sensitivity analyses, a thorough exploration of its potential sources is crucial for the appropriate interpretation of the results, assessment of evidence consistency, and guidance for future research [99, 100]. The high I^2^ values likely stem from clinical and methodological diversity across the included studies, which, although all employing multivariate logistic regression, differed in several key aspects [101].

Geographic and population diversity: The included studies spanned diverse geographic regions, including China, Japan, South Korea, the USA, the UK, and Italy (Table 1). Variations in genetic predisposition, baseline cardiovascular health profiles, prevalence of comorbidities, and perioperative care protocols between Asian and Western populations could significantly influence effect sizes [102, 103]. For instance, the relationship between age and POAF might be modulated by differing population life expectancies and comorbidity burdens. The association of male gender with POAF could vary with population-specific risk factor distributions. Subgroup analysis by region was not performed in the primary analysis due to the limited number of studies within each subgroup for most factors, but this represents a key source of the observed heterogeneity.

Temporal evolution and surgical eras: The studies were published between 2010 and 2025, encompassing nearly 15 years of advancement in thoracic surgery. During this period, there has been a marked shift from open thoracotomy to minimally invasive techniques (VATS and robotics), refinement in anesthesia management, and evolution in perioperative protocols for fluid management, analgesia, and thromboprophylaxis [103]. A study from 2010 likely reflects a different surgical and perioperative standard compared to one from 2025. This temporal shift could explain the high heterogeneity for factors like operative time (a proxy for surgical complexity and technique) and the surgical approaches themselves (VATS, Lobectomy). The effect of lymph node dissection may also have changed over time with the standardization of techniques and the adoption of selective versus systematic dissection paradigms. Furthermore, the understanding of POAF pathophysiology and associated risk factors has evolved over this period [6].

Methodological heterogeneity in POAF definition and detection: A critical source of heterogeneity likely arises from variability in how the primary outcome, POAF, was defined and monitored across studies [104]. While all studies defined POAF as new-onset AF within 30 days post-surgery, the intensity and duration of postoperative cardiac monitoring varied. Some studies relied on routine 12-lead ECGs and clinical symptoms, while others may have used continuous telemetry or Holter monitoring for several days—the period when POAF incidence peaks. This difference in monitoring intensity directly impacts detection sensitivity; studies with more intensive monitoring will identify more (often asymptomatic) POAF episodes, potentially altering the calculated strength of association for various risk factors. Similarly, the classification of AF (e.g., episode duration threshold: > 30 s vs. > 5 min) may not have been uniform. This detection bias is a well-known challenge in synthesizing POAF literature and is a plausible major contributor to the high statistical heterogeneity, particularly for patient-related factors like age and gender [105].

Variability in surgical technique and definitions: Even for ostensibly well-defined factors, clinical practice varies. Lymph node dissection can range from sampling to systematic radical dissection, with differing extents of mediastinal station clearance. Lobectomy and operative time are influenced by surgical approach (open vs. VATS), surgeon experience, and the use of advanced energy devices or staplers. This technical variability, not fully captured by the broad definitions used in observational studies, introduces “clinical noise” that manifests as statistical heterogeneity [100].

We regret that the manuscript did not include these subgroup or meta-regression analyses to quantify the contribution of these factors to heterogeneity. The primary constraint was the limited number of studies (n = 18) and the even smaller number available for each risk factor, which makes reliable subgroup analyses underpowered and unstable [106, 107]. As suggested, future updates of this meta-analysis, as more studies with multivariate adjustments are published, should prioritize pre-planned subgroup analyses or meta-regression by geographic region, surgical era (e.g., before vs. after 2015), and POAF monitoring protocol (e.g., routine ECG vs. continuous monitoring) [107]. This will be essential to move from identifying heterogeneity to explaining it, thereby offering more nuanced, context-specific risk assessments.

### Consideration of publication bias

The Egger’s and Begg’s tests indicated potential publication bias in the meta-analysis of gender (male) as a risk factor for POAF (Begg’s test P = 0.013; Egger’s test P = 0.006) [108]. We acknowledge this as an important limitation. Such bias may arise if smaller studies or those reporting non-significant findings concerning this well-established demographic factor are less likely to be published or indexed, potentially leading to an overestimation of the pooled effect size. Variability in the accessibility of studies from different regions or in different languages may also contribute to an incomplete dataset.

To assess the potential impact of this bias on our primary conclusion, we conducted sensitivity analysis using the leave-one-out method. The direction and statistical significance of the pooled OR remained stable throughout this analysis, supporting the robustness of the core finding that male gender is an independent risk factor for POAF. However, sensitivity analysis cannot fully account for bias introduced by studies missing from the literature [109]. Therefore, while the current evidence strongly supports the association, the magnitude of the effect (OR = 1.93) might be slightly overestimated. Future meta-analyses should aim to incorporate larger, high-quality studies and actively search grey literature to verify and more precisely calibrate the strength of this association.

### Consideration of omitted supplementary analyses

Regarding the assessment of dose–response relationships, our meta-analysis treated variables like age and operative time as continuous, reporting a linear OR per unit increase (Age: OR = 1.04 per year; Operative time: OR = 1.05 per minute). This provides a valid estimate of linear risk association. However, a formal non-linear dose–response meta-analysis (e.g., using restricted cubic splines) to explore potential threshold effects was not feasible. The included studies predominantly reported only a single adjusted OR for the entire continuous variable, lacking the stratified data across multiple exposure intervals required for such modeling [110].

Concerning subgroup analysis by different types within “lobectomy” (e.g., upper vs. lower lobectomy), we agree this is clinically relevant. However, across all 18 included studies, “lobectomy” was consistently reported as a binary variable (lobectomy vs. sublobar resection) in the multivariate models. None of the primary studies provided adjusted effect estimates for specific lobectomy categories, making such a subgroup analysis impossible with the available pooled data [111].

Finally, the suggestion to employ meta-regression to quantify sources of heterogeneity aligns with our qualitative discussion in Sect. "Consideration of Heterogeneity". We identified potential moderators such as geographic region and surgical era. However, the number of studies available for each key risk factor (e.g., 8 for age, 7 for lymph node dissection, 3 for lobectomy) is insufficient to support a statistically robust and reliable meta-regression analysis, which risks unstable estimates and spurious findings [112]. Therefore, we provided a comprehensive narrative synthesis of potential heterogeneity sources instead.

These omissions highlight important limitations of the current evidence base and underscore the need for future primary studies to report more granular data, enabling these advanced analyses in subsequent meta-analyses.

### Strengths and limitations

Strengths include adherence to PRISMA guidelines, systematic literature search, and restriction to multivariate-adjusted studies to minimize confounding. Comprehensive heterogeneity, sensitivity, and publication bias analyses support robust conclusions.

Limitations include the inherent inability of observational studies to prove causality and potential residual confounding. Furthermore, the assessment of continuous risk factors (e.g., age, operative time) was based on a linear per-unit increase assumption; potential non-linear dose–response relationships could not be evaluated due to insufficient data from the primary studies. Evidence of publication bias was noted for the gender analysis, which is a recognized constraint of meta-analytic methods. Some subgroups (e.g., VATS, hyperthyroidism) had few studies, limiting stability, in particular, the analysis for VATS was based on only two studies with high heterogeneity, precluding any definitive conclusions regarding its effect on POAF risk. Variable definitions and monitoring intensity for POAF across studies may introduce detection bias. We could not assess emerging biomarkers (e.g., galectin-3, GDF-15) or imaging parameters (e.g., left atrial strain) due to data availability. Furthermore, our literature search was limited to major academic databases and did not include a systematic search of grey literature. While we performed manual checks of reference lists, this approach may have resulted in the omission of unpublished or ongoing studies, which could introduce a potential source of reporting bias.

### Future research directions

Future studies should: a. Advance precision medicine: Develop machine learning-driven predictive models integrating genomics, proteomics, and imaging data [113, 114]; b. Optimize prevention: Conduct multicenter RCTs comparing tailored strategies (e.g., targeted anti-inflammatory therapy, novel antiarrhythmics, neuromodulation) [115, 116]; c. Investigate long-term outcomes: Explore POAF as a marker for future cardiovascular events and overall survival in lung cancer patients [117, 118].

## Conclusion

In summary, this meta-analysis of multivariate-adjusted studies suggests that POAF after PR is associated with a spectrum of factors, including patient susceptibility (e.g., advanced age, pre-existing AF) and surgery-related stressors (e.g., extent of resection, transfusion). Pre-existing AF was identified as the strongest predictor among the factors analyzed. The analysis did not demonstrate a significant independent association between VATS and POAF risk, although this finding is considered preliminary due to the limited number of available studies. Collectively, these findings support the rationale for a more nuanced, risk-stratified approach to perioperative management. Integrating this evidence into clinical assessment may aid in identifying high-risk patients who could benefit from intensified monitoring and personalized preventive strategies, while also highlighting critical gaps for future research.

## Supplementary Information

Additional file 1: Common techniques and categories of PR. This schematic illustrates two principal surgical approaches and various anatomical extents of PR. (**A**) Open thoracotomy, involving a large incision and rib spreading for direct visualization. (**B**) Minimally invasive VATS, performed through small incisions under video guidance. (**C**) Wedge resection: Non-anatomical removal of a small, triangular segment of lung tissue. (**D**) Segmentectomy: Anatomical resection of one or more bronchopulmonary segments. (**E**) Lobectomy: Removal of an entire lung lobe. (**F**) Pneumonectomy: Complete removal of one lung. (**G**-**H**) Bronchial sleeve resections: Resection of a bronchial segment with reconstruction to preserve distal lung function, demonstrating tumor removal along with affected bronchial sections

Additional file 2: Funnel plots for assessment of publication bias. (**A**) Age, (**B**) Gender, (**C**) Lymph Node Dissection, (**D**) VATS, (**E**) Lobectomy, (**F**) Operative Time (minutes). Each funnel plot displays the standard error (s.e.) against the OR or other effect measures, with pseudo 95% CI. Symmetrical distribution of points suggests no significant publication bias

Additional file 3: Sensitivity analysis using the leave-one-out method. (**A**) Age, (**B**) Gender, (**C**) Lymph Node Dissection, (**D**) VATS, (**E**) Lobectomy, (**F**) Operative Time. Galbraith (radial) plots show the influence of sequentially excluding individual studies on the pooled effect estimate (center dotted line) and 95% CI (vertical bars). The stability of the meta-analysis results is demonstrated by the overlapping CI across all sensitivity analyses

Additional file 4: Sensitivity analysis using the leave-one-out method to assess the influence of studies with unreported (“NR”) age data on the pooled estimate

Additional file 5:

## Data Availability

The data analyzed in this meta-analysis are derived from publicly available databases and published studies, which can be accessed through the following platforms: PubMed, Cochrane Library, Embase, WOS, CNKI, Wanfang Data, VIP Database, and Sinomed.

## Abbreviations

POAF: Postoperative atrial fibrillation
PR: Pulmonary resection
WOS: Web of science
CNKI: China national knowledge infrastructure
AF: Atrial fibrillation
PRISMA: Preferred reporting items for systematic reviews and meta-analyses
OR: Odds ratios
CI: Confidence intervals
AF: Pre-existing atrial fibrillation
VATS: Video-assisted thoracoscopic surgery
MOOSE: Meta-analysis of observational studies in epidemiology
IPRSR: International Prospective Register of Systematic Reviews
PICO: Participants, Interventions, comparisons, and outcomes
NOS: Newcastle-ottawa scale
AHRQ: Agency for healthcare research and quality
SCI: Science citation indexed
RAAS: Renin-angiotensin-aldosterone system
CRP: C-reactive protein
TRIM: Transfusion-related immunomodulation
STS: Society of thoracic surgeons
ECG: Electrocardiogram
RCTs: Randomized controlled trials
ACC/AHA: American college of cardiology / american heart association
