# Supplementary material for: Prevalence and risk factors for postoperative atrial fibrillation following pulmonary resection: a systematic review and meta-analysis
Source: J Cardiothorac Surg. 2026 Apr 18;21:318. doi: 10.1186/s13019-026-04093-x (PMC13231641; doi:10.1186/s13019-026-04093-x)
Supplement: Supplementary file 5 — Additional file 5: [file 13019_2026_4093_MOESM5_ESM.docx]

**Supplementary Tables**

**Table S1.** PubMed Retrieval strategy.

| No | Query |
| --- | --- |
| #1 | "lung surgery" |
| #2 | "Atrial Fibrillation"[Mesh] |
| #3 | ((((((((((((((((((((((((Atrial Fibrillations) OR (Fibrillation, Atrial)) OR (Fibrillations, Atrial)) OR (Auricular Fibrillation)) OR (Auricular Fibrillations)) OR (Fibrillation, Auricular)) OR (Fibrillations, Auricular)) OR (Persistent Atrial Fibrillation)) OR (Atrial Fibrillation, Persistent)) OR (Atrial Fibrillations, Persistent)) OR (Fibrillation, Persistent Atrial)) OR (Fibrillations, Persistent Atrial)) OR (Persistent Atrial Fibrillations)) OR (Familial Atrial Fibrillation)) OR (Atrial Fibrillation, Familial)) OR (Atrial Fibrillations, Familial)) OR (Familial Atrial Fibrillations)) OR (Fibrillation, Familial Atrial)) OR (Fibrillations, Familial Atrial)) OR (Paroxysmal Atrial Fibrillation)) OR (Atrial Fibrillation, Paroxysmal)) OR (Atrial Fibrillations, Paroxysmal)) OR (Fibrillation, Paroxysmal Atrial)) OR (Fibrillations, Paroxysmal Atrial)) OR (Paroxysmal Atrial Fibrillations) |
| #4 | #2 or #3 |
| #5 | "Risk Factors"[Mesh] |
| #6 | (((((((((((((((((Factor, Risk) OR (Risk Factor)) OR (Population at Risk)) OR (Populations at Risk)) OR (Risk Scores)) OR (Risk Score)) OR (Score, Risk)) OR (Risk Factor Scores)) OR (Risk Factor Score)) OR (Score, Risk Factor)) OR (Health Correlates)) OR (Correlates, Health)) OR (Social Risk Factors)) OR (Factor, Social Risk)) OR (Factors, Social Risk)) OR (Risk Factor, Social)) OR (Risk Factors, Social)) OR (Social Risk Factor) |
| #7 | #5 or #6 |
| #8 | ((lung surgery) AND (#2 or #3)) AND (#5 or #6) |

**Table S2.** Embase Retrieval strategy.

| No | Query |
| --- | --- |
| #10 | #3 AND #6 AND #9 |
| #9 | #7 OR #8 |
| #8 | ('relative risk'/exp OR 'relative risk' OR 'risk factors'/exp OR 'risk factors' OR 'risk factor'/exp OR 'risk factor') AND [embase]/lim |
| #7 | ('risk factor'/exp OR 'risk factor') AND [embase]/lim |
| #6 | #4 OR #5 |
| #5 | ('atrium fibrillation':ab,ti OR 'auricular fibrilation':ab,ti OR 'auricular fibrillation':ab,ti OR 'cardiac atrial fibrillation':ab,ti OR 'cardiac atrium fibrillation':ab,ti OR 'fibrillation, heart atrium':ab,ti OR 'heart atrial fibrillation':ab,ti OR 'heart atrium fibrillation':ab,ti OR 'heart fibrillation atrium':ab,ti OR 'non-valvular atrial fibrillation':ab,ti OR 'nonvalvular atrial fibrillation':ab,ti OR 'atrial fibrillation':ab,ti) AND [embase]/lim |
| #4 | ('atrial fibrillation'/exp OR 'atrial fibrillation') AND [embase]/lim |
| #3 | #1 OR #2 |
| #2 | ('alveolar surgery':ab,ti OR 'lung operation':ab,ti OR 'pulmonary surgery':ab,ti OR 'pulmonary surgical procedures':ab,ti OR 'surgery, lung':ab,ti OR 'lung surgery':ab,ti) AND [embase]/lim |
| #1 | ('lung surgery'/exp OR 'lung surgery') AND [embase]/lim |

**Table S3.** Publish biased assessments.

| **Outcome** | **P value for Begg test** | **P value for Egger test** |
| --- | --- | --- |
| Age | 0.805 | 0.511 |
| Gender | 0.013 | 0.006 |
| Lymph node dissection | 0.652 | 0.356 |
| VATS | 0.317 | - |
| Lobectomy | 0.602 | 0.649 |
| Operative time,mins | 0.117 | 0.254 |

Notes: VATS=Video-assisted thoracoscopic surgery.

**Table S4.** Summary of comparison with key previous meta-analyses/reviews on POAF risk factors after pulmonary resection.

| **Risk factor / Point of contention** | **Key findings (this meta-analysis)** | **Comparison with key previous evidence** |
| --- | --- | --- |
| General methodology | Included only studies employing multivariate logistic regression, providing adjusted effect estimates. | Most previous reviews were narrative or pooled unadjusted data, which are more susceptible to confounding. |
| Lymph node dissection | Identified as an independent risk factor (OR=2.18, 95% CI: 1.12-4.24). | Previous epidemiological evidence was conflicting. Our analysis of 7 studies provides a more definitive adjusted estimate. |
| Surgical approach (VATS) | No significant independent association was found (OR=1.02, 95% CI: 0.29-3.61, p=0.97). | Earlier studies suggested VATS might reduce POAF. Recent high-quality propensity-matched studies support our conclusion of no independent protective effect after adjusting for confounders. |
| Age & Gender | Age (per year): OR=1.04; Age ≥65 years: OR=2.92. Male: OR=1.93. | Findings are consistent with large database analyses (e.g., STS). Our study further quantifies the risk gradient for continuous age and provides a pooled adjusted estimate for males. |
| History of hyperthyroidism | Identified as a strong risk factor (OR=5.67, 95% CI: 1.97-16.32). | As a rare but potent factor, our study provides a quantitative, pooled effect estimate that underscores its clinical importance. |
| Risk factor categorization | Proposed a descriptive categorization (“Very strong, Strong, Moderate, Low”) based on explicit OR thresholds. | Offers a clearer, quantitative hierarchy of risk to facilitate quick clinical interpretation and risk stratification. |

Notes: POAF = Postoperative Atrial Fibrillation; VATS=Video-assisted thoracoscopic surgery.
